# Take a step back to see your own value: on the role of metacognition in self-esteem regulation

**DOI:** 10.3389/fpsyg.2025.1530008

**Published:** 2025-03-14

**Authors:** Lena Rader, Saskia Doreen Forster, Siegfried Gauggel, Barbara Drueke, Verena Mainz

**Affiliations:** Institute of Medical Psychology and Medical Sociology, University Hospital RWTH Aachen, Aachen, Germany

**Keywords:** self-esteem, decentering, self-affirmation, self-protection, self-enhancement

## Abstract

**Introduction:**

When self-esteem is threatened (e.g., by social rejection), people regulate it through self-enhancement, self-protection, or self-affirmation. High self-esteem individuals use functional strategies like self-affirmation and self-enhancement, while those with low self-esteem rely more on self-protection strategies. This study explored whether decentering, a metacognitive process, aids in accessing resources and promoting functional self-esteem regulation.

**Methods:**

1,100 participants (age 18–65, 72% female) completed questionnaires online. Structural equation modeling was used to test whether decentering mediates the association between self-esteem and self-enhancement, self-affirmation and self-protection.

**Results:**

Self-esteem positively predicted decentering, which promoted self-affirmation and self-enhancement. The decentering factor *Accepting Self-perception* positively predicted self-protection, while the *Distanced Perspective* factor reduced it. Decentering significantly mediated all three strategies.

**Discussion:**

These findings suggest that enhancing decentering could improve self-esteem regulation and inform therapeutic interventions. Strengthening an accepting self-perception may help individuals with low self-esteem adopt protective strategies. Fostering a distanced perspective could further promote self-affirmation, leading to better mental health outcomes.

## Introduction

In everyday life, it often happens that one is criticized or questioned in some way, which can pose a threat to one’s self-esteem. According to Baumeister et al. (1989), self-esteem describes the extent to which a person values him- or herself and is largely formed through the reciprocity between the self and social interaction (Leary, 2005). A person with a high self-esteem is supposed to have experienced more positive social interactions and might be convinced to have many positive attributes. In contrast, a person with a low self-esteem might show the opposite pattern (Baumeister et al., 1989). As a high self-esteem is typically associated with higher psychological well-being (Du et al., 2017; Lannin et al., 2021), low self-esteem, on the other hand, seems to constitute a risk factor for the development of mental illnesses such as depression (e.g., Orth and Robins, 2013). Although self-esteem is traditionally measured as a stable construct, we now know that it is susceptible to social rejections or approval and can fluctuate across situations (Lannin et al., 2021; Leary, 2005).

The sociometer theory by Leary et al. (1995) emphasizes the importance of social interactions in maintaining a high self-esteem and posits that people have a fundamental need to belong. In this framework, the self-esteem serves as an index of a person’s perceived level of social acceptance and can thus be considered as sociometer (Leary, 2005). For instance, following social approval like a compliment, a person might feel highly accepted and his or her self-esteem is boosted. Social rejections, on the other hand, are rather associated with negative emotions as well as a lowered self-esteem. Interestingly, Leary (2005) found a positive association between social rejections and negative emotions and a negative association between a person’s stable (trait) self-esteem and negative emotions following social rejections. These findings suggest two important implications: First, people generally tend to experience negative emotions and a drop in self-esteem after social rejections. Second, people with a low trait self-esteem experience even more negative emotions following a rejection compared to individuals with a high trait self-esteem. A person’s trait self-esteem thus predicts the sensitivity of the sociometer such that persons with low trait self-esteem might be especially sensitive to social rejections. This is in line with the results from Murray et al. (2002) revealing that individuals with lower trait self-esteem are more prone to feeling threatened in social interactions than those with higher trait self-esteem. This was indicated by a significant interaction between acceptance threat (e.g., leading participants to believe that their partner was annoyed or troubled by some aspect of their personality) and trait self-esteem. More specifically, individuals with low trait self-esteem felt significantly less accepted by their partner when facing an acceptance threat compared to those with high trait self-esteem. People with low trait self-esteem might thus have a miscalibrated sociometer caused by a chronic inclusion deficit (Murray et al., 2002).

Given that people generally tend to strive to maintain high self-esteem, a threat to self-esteem (for example, through criticism) can lead to cognitive dissonance (Alicke and Sedikides, 2009; Steele, 1988) which in turn is difficult to manage and requires the use of regulatory strategies in order to resolve it. The sociometer theory describes three main regulatory strategies: self-enhancement, self-affirmation and self-protection (Leary, 2007). Numerous studies have emphasized the importance of these regulative strategies, also referred to as motivational tendencies, in the maintenance of self-esteem (Alicke and Sedikides, 2009; Hepper et al., 2010). Self-enhancement is a habitual strategy that people use to bolster their self-esteem and describes the tendency to view oneself in a positive light and to embrace social approval (e.g., compliments; Hepper et al., 2010). In contrast, self-affirmation and self-protection are described as situational strategies that are used following threats to self-esteem such as a negative feedback (Harris et al., 2019; Hepper et al., 2010). Steele (1988) observed early on that people employ various strategies to resolve cognitive dissonance, such as rationalization, external attribution of failure, or even dampening negative emotions with alcohol or drugs. This cognitive modification of negative, self-threatening information is referred to as self-protection (for example, derogating the person who gave a negative feedback; Hepper et al., 2010). However, Steele (1988) also found that people did not feel the urge to use these defensive strategies under certain conditions, namely when they were given the opportunity to recall something personally relevant to them, such as their own values and principles. Steele (1988) termed this strategy self-affirmation. Through self-affirmation, individuals can restore their global self-worth and reassure themselves of their moral adaptiveness by focusing on something important to them such as personal values or significant social relationships, acts of kindness or generosity, and recalling personal resources such as strengths, positive traits, skills, and accomplishments (Harris et al., 2019; MCQueen and Klein, 2006; Steele, 1988).

Numerous studies have demonstrated the positive impact of all three strategies on self-esteem and psychological well-being (e.g., Alicke and Sedikides, 2009; Hepper et al., 2010; Luo et al., 2020). However, self-affirmation stands out as the only approach that not only promotes well-being but also empowers individuals to face threatening information—such as criticism—openly, thereby fostering opportunities for personal growth (Harris et al., 2019). In contrast, some studies found a link between self-enhancement and narcissistic personality traits and self-serving distortions of reality to enhance self-esteem (Grijalva and Zhang, 2016). Studies also suggest that individuals who employ self-protection strategies after being self-threatened typically react defensively and deny the self-threatening information (Wullenkord and Reese, 2021). However, when they use self-affirmation strategies, it may help them to approach this information more openly (Harris et al., 2019). For instance, a study by Düring and Jessop (2015) demonstrates that individuals who were given the opportunity to reflect on personally important values (self-affirmation intervention) before receiving health-threatening information (self-esteem threat) exhibited lower message derogation, more positive attitudes, and higher motivation to change their behavior compared to those who did not receive a self-affirmation intervention. Self-affirmation has also been linked to more systematic processing and higher open-mindedness toward self-threatening information (Harris et al., 2019). All in all, these results indicate that self-affirmation can help individuals in the regulation of their self as a whole in a functional way.

Interestingly, one’s self-esteem is one factor that can explain why some individuals are more inclined to use self-enhancement, self-protection, or self-affirmation strategies. While self-enhancement and self-affirmation are positively associated with self-esteem, self-protection shows a negative correlation with self-esteem (Harris et al., 2019; Hepper et al., 2010). Studies such as Harris et al. (2019) suggest that individuals with high self-esteem are better able to access their resources, allowing them to utilize more adaptive strategies like self-affirmation which aligns with the Affirmational Resources View by Steele et al. (1993). Rader et al. (2024) further suggest that the Affirmational Resources View might primarily concern self-affirmation using internal resources such as one’s strengths and personal values. In contrast, external resources (e.g., friends and family) might be equally accessible to both high and low self-esteem individuals (Rader et al., 2024). Given the benefits of self-affirmation for well-being, further research should focus on promoting its use, especially for individuals with low self-esteem, as they are more prone to negative emotions and mental health risks. This study aims to explore factors influencing individual differences in the use of self-affirmation, self-enhancement, and self-protection strategies.

In our study, we propose decentering as one such factor. Decentering is a meta-cognitive process that allows an individual to view information from a distanced perspective without emotional judgment (Fresco et al., 2007a,b; Safran and Segal, 1996). This seems to be particularly important in the context of processing self-threatening information: a shift from evaluative processing of the threatening information to non-judgmental awareness through decentering may allow individuals a more open-minded processing of self-threatening information. At the same time, decentering has been shown to promote functional self-focused attention (Kessel et al., 2016). Numerous studies have also demonstrated positive effects of decentering on mental health in both the general population and patients with mental illnesses (Hayes-Skelton and Lee, 2018; Mainz et al., 2016; Moore et al., 2022; O’Toole et al., 2019). Therefore, decentering has become an important component in the treatment of depression and anxiety disorders such as Mindfulness-Based Cognitive Therapy (Bennett et al., 2021; Segal et al., 2018). Bernstein et al. (2015, 2019) proposed a metacognitive process model of decentering which defines three components that influence each other and collectively describe decentering. The first component is *meta-awareness*, which describes the awareness that the experience of a moment is subjective. The second component is *disidentification from internal experience* which is the „experience of internal states as separate from one’s self “(Bernstein et al., 2015, p. 3). The third component is *reduced reactivity to thought content*; it means that negative thoughts or memories do not lead to a worsening of mood and influence other cognitive processes such as attention.

In contrast, the data-driven account, which involves factor analyses conducted with various decentering questionnaires, suggests two components of decentering (Bennett et al., 2021). The first factor, for example, referred to as *Intentional Decentered Perspective* (Hadash et al., 2017), *Observer Perspective* (Naragon-Gainey and DeMarree, 2017) or *Distanced Perspective* (Gecht et al., 2014; Rader et al., 2023), collectively describes the ability to take a distant perspective on negative events (e.g., thoughts, memories). The second factor found across decentering questionnaires has been referred to as *Automatic Reactivity to Thought Content* (Hadash et al., 2017), *Reduced Struggle* (Naragon-Gainey and DeMarree, 2017) or *Accepting Self-perception* (Gecht et al., 2014; Rader et al., 2023) and describes the associated reduced emotional reaction to these negative events. In the present study, we utilize the data-driven two-factorial decentering model with the *Distanced Perspective* and *Accepting Self-perception* factors proposed by Gecht et al. (2014) and Rader et al. (2023). It should be noted that the *Accepting Self-perception* factor was found to be significantly stronger correlated with self-esteem than the *Distanced Perspective* factor and likely encompasses aspects of self-compassion in addition to decentering (Rader et al., 2023). The finding that decentering and self-esteem are related, and the fact that decentering is a process, leads us to the question of whether decentering also influences the choice of strategies for self-esteem regulation. To the best of the authors’ knowledge, while these models are widely used in numerous studies (e.g., Bernstein et al., 2019; Hanley et al., 2020), they have not yet been examined in connection with self-esteem regulation which is the aim of the current study.

We hypothesize that decentering mediates the known association between self-esteem and the regulative strategies self-affirmation, self-enhancement, self-protection (hypothesis 1). More specifically, we expect a positive link between self-esteem and decentering which has already been demonstrated in previous studies - whereby the *Accepting Self-perception* factor should correlate more strongly with self-esteem than the *Distanced Perspective* factor (e.g., Rader et al., 2023; hypothesis 1.1). It is assumed that decentering enables a functional self-focused attention, allowing individuals to better access their resources when facing threats to their self-esteem (such as criticism). This would in turn enable them to employ self-affirmation strategies (rather than self-protection) to regulate their self-esteem. Critcher and Dunning (2015) already postulated the idea that the positive effects of self-affirmation can be explained by a broadening of perspective. After a threat to self-esteem, the threatened part of the self becomes highly salient (active working memory), and the rest of the self is no longer accessible. According to Critcher and Dunning (2015), self-affirmation enables a broadening of perspective, allowing the threat to be viewed as separate from the self. A study by vandellen et al. (2012) further illustrates the connection between self-esteem and information processing or attentional focus after a threat to self-esteem. They found that participants with low self-esteem directed their attention more toward their environment and social cues rather than themselves following social rejections. After the threat, attention was narrowed (particularly for individuals with low self-esteem), and active working memory was restricted to the threatening information. In light of the sociometer theory, the results by vandellen et al. (2012) could be interpreted as follows: individuals with low self-esteem, due to (anticipated) repeated experiences of exclusion, may possess a miscalibrated sociometer and thus react more sensitively to social rejections. This miscalibrated sociometer could lead to directing attention toward the environment and social cues in preparation for potential rejection. In contrast, individuals with higher self-esteem may be more capable of directing attention toward themselves in a functional manner (more decentering), enabling them better access to their resources and thus facilitating the use of self-affirmation (rather than self-protection) strategies.

**Figure 1:**
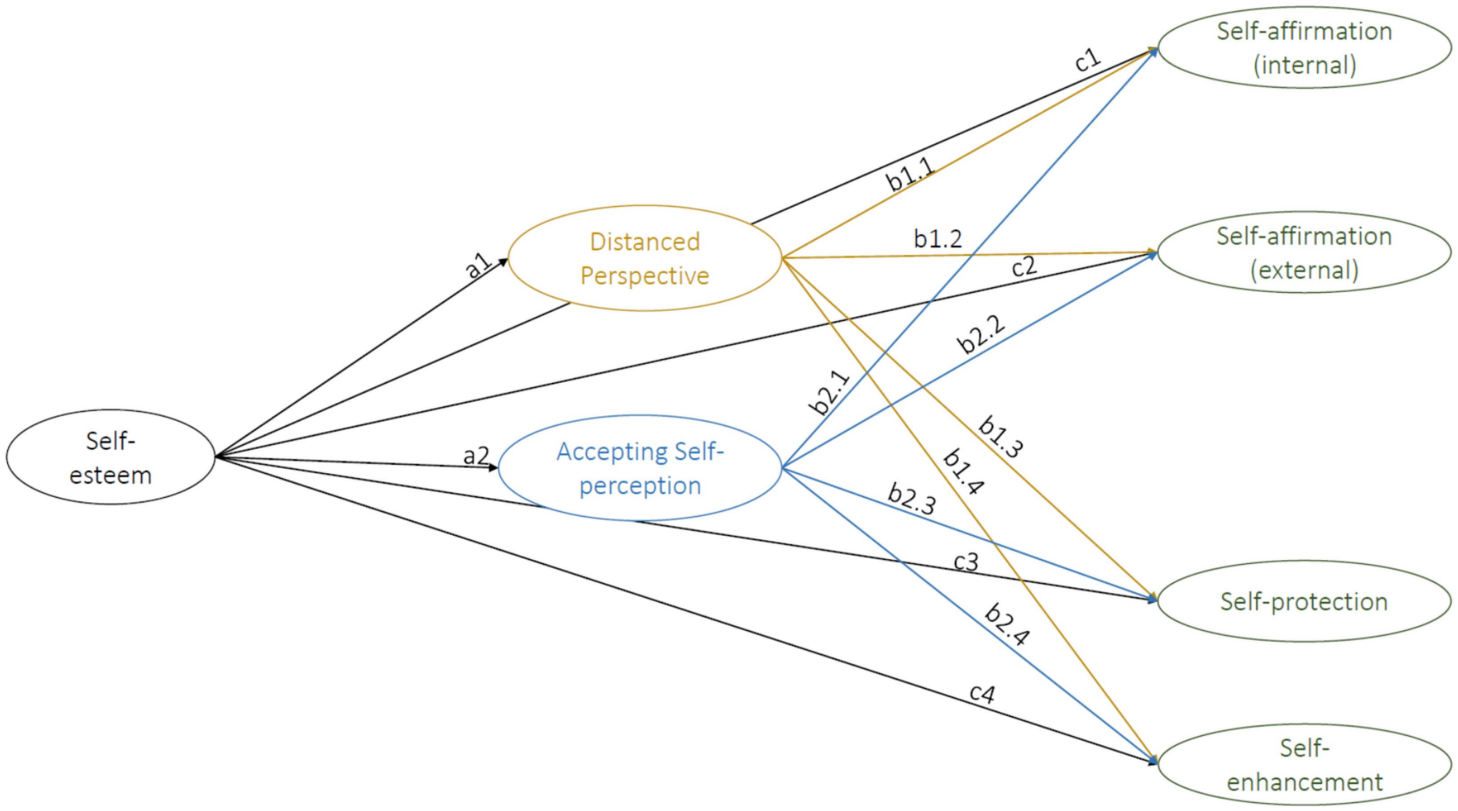
Overview of variables and paths included in the structural equation model (hypothesis 1).

In summary, research suggests that low self-esteem is linked to heightened sensitivity to social rejection, often accompanied by defensive self-protection strategies due to limited awareness of one’s own resources. In contrast, individuals with high self-esteem tend to adopt self-affirmation strategies instead. Additionally, there is evidence that contemplative practices, such as mindfulness – a concept closely related to decentering (Hadash et al., 2017) – can mitigate defensiveness in response to self-threats by quieting the ego (Arahuete and Pinazo, 2024). In the present study, we propose that decentering reduces defensiveness through its “ego quieting” effect, which could be attributed to a distanced, self-accepting perspective and enhanced access to personal resources. Thus, the present study fills an important gap in the research by explicitly examining the role of decentering in self-esteem regulation. Based on findings described above and the positive associations between self-affirmation and open-mindedness and systematic processing (Harris et al., 2019), we hypothesize a positive association between decentering and self-affirmation (hypothesis 1.2). Given the expected positive association between self-esteem and decentering, a stronger association between decentering and internal self-affirmation resources compared to external self-affirmation resources is anticipated. We also hypothesize a positive link between decentering and self-enhancement (hypothesis 1.3). Finally, we expect a negative association between decentering and self-protection (hypothesis 1.4). An overview of all hypotheses of the present study is provided in Table 1. Figure 1 depicts the corresponding structural equation model with all variables of the current study and their respective paths (which are also listed in Table 1).

**Table 1:** Overview of the pre-registered hypotheses in the present study with reference to the respective paths in the structural equation model (Figure 1).

Hypothesis		Path
1	The relationship between self-esteem and the regulative strategies self-enhancement, self-protection and self-affirmation is mediated by decentering.	a1*b1.1, a1*b1.2, a1*b1.3, a1*b1.4, a2*b2.1, a2*b2.2, a2*b2.3, a2*b2.4
1.1	Self-esteem positively predicts decentering.	a1, a2
1.2	Decentering positively predicts self-affirmation.	b1.1, b1.2, b2.1, b2.2
1.3	Decentering positively predicts self-enhancement.	b1.4, b2.4
1.4	Decentering negatively predicts self-protection.	b1.3, b2.3
2.1	Self-affirmation positively predicts perceived social support.	
2.2	Self-enhancement positively predicts perceived social support.	
2.3	Self-protection negatively predicts perceived social support.	

In addition, next to the effect of decentering on self-esteem strategies (self-affirmation, self-enhancement, and self-protection), we aim to examine the adaptiveness of these strategies. As previously described, self-affirmation has been depicted as highly adaptive strategy (e.g., Düring and Jessop, 2015). Cohen and Sherman (2014) elucidate a cycle of adaptive potential in their work, which is triggered by self-affirmation. According to the authors, self-affirmation initiates a “positive feedback loop between the self-system and the social system that propagates adaptive outcomes over time” (Cohen and Sherman, 2014, p. 335). The adaptiveness of the strategies will be operationalized as perceived social support in the current study. We hypothesize that the strategies that are positively associated with self-esteem and decentering (self-affirmation and self-enhancement) positively predict perceived social support (hypothesis 2.1 and 2.2). In contrast, self-protection, which is expected to be negatively associated with self-esteem and decentering is expected to negatively predict perceived social support (hypothesis 2.3).

## Methods

### Participants

In total, 1,100 participants were recruited online. Participants of the general population were recruited from the Qualtrics participant pool in the UK (*n* = 550) and the USA (*n* = 550). The mean age was 47 years (SD = 12) ranging from 18 to 65 years and 72% of the participants identified as female. The school degrees were distributed as follows: 51% high school degree, 41% university degree, 2% middle school degree, and 6% another degree. All hypotheses and analyses conducted in the current study were pre-registered unless otherwise indicated (https://doi.org/10.23668/psycharchives.5105). We report all manipulations, measures, and exclusions applied in the current study.

### Materials

#### Decentering

Decentering was assessed using the Experiences Questionnaire (EQ) by Fresco et al. (2007a). It is comprised of 14 decentering and 6 rumination items. The items are answered on a scale from 1 (Never) to 5 (All the time). We used the factor structure proposed by Gecht et al. (2014) and Rader et al. (2023) with a *Distanced Perspective* (item 16, 17, 18 and 20) and an *Accepting Self-perception* (item 3, 5, 6, 8, 9, 10 and 15) factor. Both factors showed a high reliability in the current study (*Distanced Perspective*: *ω* = 0.74; *Accepting Self-perception*: ω = 0.86).

#### Self-esteem

The Rosenberg Self-esteem Scale (RSES; Rosenberg, 1965) was used to as self-esteem measure. Items are answered on a scale from 1 (Strongly disagree) to 4 (Strongly agree). The items 1, 3, 4, 7, and 10 were reverse-coded so that a high score indicates high self-esteem. The RSES has good internal consistency (*α* = 0.86; Tinakon and Nahathai, 2012; current study: *ω* = 0.94).

#### Self-affirmation

Self-affirmation strategies were assessed using the Spontaneous Self-Affirmation Measure (SSAM) by Harris et al. (2019) that is comprised of 13 items answered on a scale from 1 (completely disagree) to 7 (completely agree). We used the factor structure found in Rader et al. (2024) which classifies the three self-affirmation factors *Strengths*, *Values* and *Social relations* proposed by Harris et al. (2019) into *Internal resources* (Strengths, item 1, 8, 9 and 13; Values, item 2, 3, 5 and 12) and *External resources* (item 4, 6, 7, 10, 11). Both the *Internal resources* (*ω* = 0.82) and the *External resources* factor showed a high reliability in the current study (*ω* = 0.92).

#### Self-enhancement and self-protection

The Self-Enhancement Self-Protection Strategies Scale (SESP) by Hepper et al. (2010) was used to assess self-enhancement and self-protection strategies. The SESP is comprised of 60 items in total. However, only self-enhancement (favorable construals, positivity embracement) and self-protection (defensiveness) items were used in the current study. The items are answered on a scale from 1 (not at all characteristic of me) to 6 (very characteristic of me). All three scales showed a high reliability in two studies by Hepper et al. (2010); defensiveness: *α* = 0.83–0.86; favorable construals: α = 0.69–0.74; positivity embracement: α = 0.78. In the current study, we found a high internal consistency for the self-enhancement items (favorable construals, positivity embracement; *ω* = 0.89) and the self-protection items (*ω* = 0.89).

#### Social support

The adaptiveness of the self-regulative strategies was assessed using the English short version of the perceived social support questionnaire (F-Sozu K6; Lin et al., 2019). It is comprised of 6 items that are answered on a scale from 1 (not at all true) to 5 (very true). The F-Sozu K6 showed a high reliability in different samples (*α* = 0.90–0.94; Lin et al., 2019; current study: *ω* = 0.88).

### Procedure

Participants answered the questionnaires online via the platform Qualtrics. The participants first answered sociodemographic questions regarding their age, gender, spoken language, nationality, and school degree. Participants who did not indicate that they are fluent in English or were younger than 18 years or older than 65 years were automatically screened out. Next, the participants answered the questionnaires in randomized order to avoid transfer effects. To assess the data integrity, two attention checks were integrated in the questionnaires asking participants to choose the answer option “all the time” for this item. Participants who did not pass both attention checks were automatically screened out. At the end of data collection, another data integrity check was conducted screening for participants who had a variance of zero across all items of a questionnaire. However, no participant had to be excluded based on this check.

### Data analysis

We used R for all analyses (R Core Team, 2022). The code and data behind all analyses have been made publicly available at Open Science Framework.^1^ Hypothesis 1 was tested using a structural equation model. Before calculating the structural equation model, we conducted two non-preregistered preliminary analyses to check if previous findings on the associations between self-esteem and decentering and self-esteem and the strategies could be replicated and also to get a first idea about the associations between the strategies and decentering. For this purpose, we assessed the correlations between the scale means of the decentering factors *Accepting Self-Perception* and *Distanced Perspective* from the EQ, the rumination items from the EQ, the *Internal resources* and *External resources* factors from the SSAM, the self-enhancement and self-protection factors from the Self-enhancement Self-protection and the Rosenberg Self-esteem Scale using Pearson’s r. Since we did not find the expected negative association between self-esteem and self-protection that is described in the literature, we also conducted analyses of variance on the effect of self-esteem for each strategy that can be found in the Supplementary material 1.

To test the hypothesis that decentering mediates the association between self-esteem and the regulative strategies (hypothesis 1), we used maximum likelihood as estimation method for the structural equation model. The results of the $\chi^{2}$ -test and the $\chi^{2}$ /df ratio as well as CFI (> 0.95, Jackson et al., 2009) and RMSEA (< 0.06, Jackson et al., 2009) will be reported as fit indices for the measurement part of the mediation analysis. Next, the structural part of the model for hypothesis 1 was defined. Self-esteem was entered as predictor variable, self-affirmation, self-protection and self-enhancement as criterion variables, and the decentering factors *Distanced Perspective* and *Accepting Self-perception* as mediator variable (see Figure 1). Mediation was established using the steps suggested by Zhao et al. (2010). According to the authors, mediation can be assumed when the indirect path (a*b) is significant. Indirect-only (full) mediation is assumed when only the indirect (a*b) but not the direct (c) path is significant. If both the indirect and the direct path are significant, partial (complementary or competitive) mediation can be assumed. The significance of the indirect path was tested using a bootstrap test with maximum likelihood estimation and 1,000 iterations (Preacher and Hayes, 2004). The structural equation model was estimated using the lavaan package (Rosseel, 2012). Hypothesis 2 was also tested using a structural equation model. For the structural part, perceived social support was added as criterion variable and self-affirmation (*Internal resources, External resources*), self-enhancement and self-protection as predictor variables.

After reviewing the hypotheses, an additional non-preregistered post-hoc network analysis was conducted to further examine the previously unknown relationships between decentering and the strategies in more detail. The aim of the network analysis was to investigate possible central strategies that, for example, are particularly strongly related to the decentering factors *Distanced Perspective* or *Accepting Self-perception*. Network analyses are now a widely used method for examining psychological constructs, where all variables are treated as observed variables (instead of latent variables as in classic test theory; Borsboom, 2017). The network is based on a partial correlation matrix, to which a tuning parameter (Least Absolute Shrinkage and Selection Operator, LASSO) is applied to reduce spurious correlations. The result is a sparse (partial) correlation matrix. LASSO produces multiple networks with varying degrees of conservative tuning parameters. To determine the best tuning parameter (the sparseness of the correlation matrix), the Extended Bayesian Information Criterion (EBIC) can be used (preferring the model with the lowest EBIC). The sparse partial correlation matrix is then graphically represented (glasso), where nodes represent the questionnaire items and edges represent the partial correlations between items. Positive correlations are represented by blue edges and negative correlations by red edges. The thicker the edge weight, the larger the correlation (Hevey, 2018). In this post-hoc analysis, three networks were estimated using the sum scores of the decentering factors *Accepting Self-perception* and *Distanced Perspective* and the individual items for self-protection, self-enhancement, and self-affirmation. For each network, the centrality measures strength and betweenness were determined. Strength is derived from the sum of all edge weights and represents the total number of connections a node has within the network. Betweenness indicates how often a node lies on the path between two other nodes, essentially showing if it acts as a mediator or intermediary between two other nodes (Hevey, 2018). The network analyses were conducted using the qgraph package (Epskamp et al., 2012).

## Results

The preliminary correlation analyses with the mean scores of the questionnaires showed that the decentering factors *Accepting Self-Perception* and *Distanced Perspective* correlated most strongly with the internal self-affirmation resources (DP: *r* = 0.47, *p* < 0.01; AS: *r* = 0.51, *p* < 0.01;), followed by self-enhancement (DP: *r* = 0.43, *p* < 0.01; AS: *r* = 0.47, *p* < 0.01) and then external self-affirmation resources (DP: *r* = 0.31, *p* < 0.01; AS: *r* = 0.27, *p* < 0.01). For self-protection, a very weak but still significant positive correlation with self-esteem was found (DP: *r* = 0.08, *p* < 0.01; AS: *r* = 0.14, *p* < 0.01). On the other hand, when looking at the correlations with the rumination items of the EQ, self-protection was the only strategy significantly positively associated with rumination (*r* = 0.13, *p* < 0.01). The results already broadly indicate that self-affirmation is more strongly related to the meta-cognitive process of decentering, whereas self-protection strategies are more likely to be associated with rumination. The results of the correlation analyses as well as the means and standard deviations of the study constructs are shown in Table 2.

**Table 2:** Results of the preliminary correlation analyses and means and standard deviations of the study constructs.

	Mean	SD	1	2	3	4	5	6	7	8
1 Sa (int.)	4.43	1.35	–							
2 Sa (ext.)	5.05	1.34	0.65*	–						
3 Se	3.53	0.76	0.61*	0.38*	–					
4 Sp	2.66	0.77	0.25*	0.14*	0.55*	–				
5 DP	3.44	0.69	0.47*	0.31*	0.43*	0.08*	–			
6 AS	2.91	0.69	0.51*	0.27*	0.47*	0.14*	0.57*	–		
7 Rum	3.55	0.62	−0.04	0.03	0.04	0.13*	0.05	−0.32*	–	
9 Rses	2.75	0.64	0.55*	0.27*	0.47*	0.03	0.48*	0.64*	−0.37*	–

Sa (int.) = Self-affirmation (internal resources), Sa (ext.) = Self-affirmation (external resources), Se = Self-enhancement, Sp = Self-protection, DP = Distanced Perspective, AS = Accepting Self-perception, Rum = Rumination, Rses = Rosenberg Self-esteem Scale, *p < 0.01.

Next, the results of the structural equation model will be reported. The model (shown in Figure 1) fit the data acceptably based on model fit indices (*χ*^2^ (1806) = 7490.65, *p* < 0.001, χ^2^/df = 4.15, CFI = 0.838, RMSEA = 0.054). Regarding hypothesis 1.1, we found that both the *Accepting Self-perception* (*β* = 0.97, SE = 0.06, 95% CI [0.85, 1.09], *p* < 0.001) and the *Distanced Perspective* (*β* = 0.61, SE = 0.05, 95% CI [0.52, 0.70], *p* < 0.001) factor of the EQ were significantly positively predicted by self-esteem.

### Self-affirmation

#### Internal resources

Both the *Accepting Self-perception* factor (*β* = 0.32, SE = 0.11, 95% CI [0.11, 0.53], *p* < 0.001) and the *Distanced Perspective* factor (*β* = 0.54, SE = 0.12, 95% CI [0.33, 0.78], *p* < 0.001) significantly positively predicted self-affirmation with internal resources (hypothesis 1.2). The results of the mediation analysis revealed that the direct effect of self-esteem on self-affirmation using internal resources (*β* = 0.67, SE = 0.13, 95% CI [0.41, 0.93], *p* < 0.001) was mediated by the *Accepting Self-perception* factor (*β* = 0.31, SE = 0.11, 95% CI [0.11, 0.54], *p* < 0.01) and the *Distanced Perspective* factor (*β* = 0.32, SE = 0.07, 95% CI [0.20, 0.48], *p* < 0.01), as evidenced by significant indirect effects. These findings suggest that decentering plays a significant role in explaining the relationship between self-esteem and self-affirmation using internal resources, supporting the hypothesized mediation pathway. The total effects of self-esteem and decentering on self-affirmation using internal resources were also positive and significant (AS: *β* = 0.98, SE = 0.10, 95% CI [0.81, 1.19], *p* < 0.01; DP: *β* = 0.99, SE = 0.13, 95% CI [0.74, 1.24], *p* < 0.01).

#### External resources

Self-affirmation using external resources was significantly positively predicted by the *Distanced Perspective* factor (*β* = 0.61, SE = 0.13, 95% CI [0.38, 0.89], *p* < 0.01) but not the *Accepting Self-perception* factor (*β* = 0.13, SE = 0.12, 95% CI [−0.10, 0.38], *p* = 0.30; hypothesis 1.2). The relationship between self-affirmation (external resources) and self-esteem was fully mediated by the *Distanced Perspective* factor, as only the indirect (*β* = 0.37, SE = 0.08, 95% CI [0.22, 0.54], *p* < 0.01) but not the direct path (*β* = 0.13, SE = 0.16, 95% CI [−0.17, 0.45], *p* = 0.40) were significant. The indirect path for the *Accepting Self-perception* factor was not significant (*β* = 0.12, SE = 0.12, 95% CI [−0.10, 0.36], *p* = 0.30). Hence, the relationship between self-esteem and self-affirmation with external resources is fully mediated by the decentering factor *Distanced Perspective* factor, but not by the *Accepting Self-perception* factor. The total effects of self-esteem and decentering on self-affirmation using external resources were both positive and significant (DP: *β* = 0.50, SE = 0.15, 95% CI [0.23, 0.81], *p* < 0.01); (AS: *β* = 0.26, SE = 0.11, 95% CI [0.05, 0.49], *p* = 0.02).

### Self-enhancement

Self-enhancement was significantly positively predicted by the *Accepting Self-perception* (*β* = 0.24, SE = 0.07, 95% CI [0.10, 0.39], *p* < 0.01) and *Distanced Perspective* (*β* = 0.39, SE = 0.08, 95% CI [0.24, 0.55], *p* < 0.01) factors (hypothesis 1.3). The direct path between self-esteem and self-enhancement (*β* = 0.34, SE = 0.10, 95% CI [0.16, 0.56], *p* < 0.01) was mediated by both decentering factors (AS: *β* = 0.23, SE = 0.07, 95% CI [0.09, 0.38], *p* < 0.01; DP: *β* = 0.24, SE = 0.05, 95% CI [0.15, 0.34], *p* < 0.01). The total effect of self-esteem and decentering on self-enhancement was significant and positive (AS: *β* = 0.57, SE = 0.08, 95% CI [0.42, 0.74], *p* < 0.01; DP: *β* = 0.58, SE = 0.10, 95% CI [0.39, 0.78], *p* < 0.01).

### Self-protection

Self-protection was significantly positively predicted by the *Accepting Self-perception* factor (*β* = 0.21, SE = 0.07, 95% CI [0.08, 0.36], *p* < 0.01). The *Distanced Perspective* factor negatively though non-significantly predicted self-protection (*β* = −0.03, SE = 0.05, 95% CI [−0.13, 0.07], *p* = 0.55; hypothesis 1.4). The direct path between self-esteem and self-protection (*β* = −0.22, SE = 0.08, 95% CI [−0.39, −0.07], *p* < 0.01) was mediated by the *Accepting Self-perception* factor (*β* = 0.20, SE = 0.07, 95% CI [0.08, 0.35], *p* < 0.01) as evidenced by a significant indirect effect, but not by the *Distanced Perspective* factor (*β* = −0.02, SE = 0.03, 95% CI [−0.08, 0.04], *p* = 0.55). We thus found a negative association self-esteem and self-protection (direct path) and a positive association between self-protection and the *Accepting Self-perception* factor but no significant association with the *Distanced Perspective factor*. The link between self-esteem and self-protection was only significantly mediated by the *Accepting Self-perception* factor. Hypothesis 1.4 could thus not be confirmed. The total effect of self-esteem and the *Distanced Perspective* factor on self-protection was negative and significant (*β* = −0.24, SE = 0.08, 95% CI [−0.41, −0.09], *p* < 0.01). The total effect of self-esteem and the *Accepting Self-perception* factor on self-protection was not significant (*β* = −0.02, SE = 0.04, 95% CI [−0.10, 0.07], *p* = 0.65). Table 3 shows all relevant path coefficients that were estimated in the mediated structural equation model.

**Table 3:** Variables and paths of the structural equation model with mediation and corresponding regression coefficient with 95% confidence interval.

Criterion variable	Predictor variable	Path	Est	95% CI		SE	*b*	*p*
				Lower	Upper			
DP	Se	a1	0.61	0.52	0.70	0.05	0.62	< 0.01
AS	Se	a2	0.97	0.85	1.09	0.06	0.78	< 0.01
Self-affirmation (internal resources)	Se	c1	0.67	0.41	0.93	0.13	0.33	< 0.01
	DP	b1.1	0.54	0.33	0.78	0.12	0.26	< 0.01
	AS	b2.1	0.32	0.11	0.53	0.11	0.20	< 0.01
		a1*b1.1	0.32	0.20	0.48	0.07	0.16	< 0.01
		a2*b2.1	0.31	0.11	0.54	0.11	0.15	< 0.01
		c1 + a1*b1.1	0.99	0.74	1.24	0.13	0.49	< 0.01
		c1 + a2*b2.1	0.98	0.81	1.19	0.10	0.48	< 0.01
Self-affirmation (external resources)	Se	c2	0.13	−0.17	0.45	0.16	0.06	0.40
	DP	b1.2	0.61	0.38	0.89	0.13	0.28	< 0.01
	AS	b2.2	0.13	−0.10	0.38	0.12	0.08	0.30
		a1*b1.2	0.37	0.22	0.54	0.08	0.18	< 0.01
		a2*b2.2	0.12	−0.10	0.36	0.12	0.06	0.30
		c2 + a1*b1.2	0.50	0.23	0.81	0.15	0.24	< 0.01
		c2 + a2*b2.2	0.26	0.05	0.49	0.11	0.12	0.02
Self-protection	Se	c3	−0.22	−0.39	−0.07	0.08	−0.26	< 0.01
	DP	b1.3	−0.03	−0.13	0.07	0.05	−0.03	0.55
	AS	b2.3	0.21	0.08	0.36	0.07	0.30	< 0.01
		a1*b1.3	−0.02	−0.08	0.04	0.03	−0.02	0.55
		a2*b2.3	0.20	0.08	0.35	0.07	0.23	< 0.01
		c3 + a1*b1.3	−0.24	−0.41	−0.09	0.08	−0.28	< 0.01
		c3 + a2*b2.3	−0.02	−0.10	0.07	0.04	−0.02	0.65
Self-enhancement	Se	c4	0.34	0.16	0.56	0.10	0.25	< 0.01
	DP	b1.4	0.39	0.24	0.55	0.08	0.29	< 0.01
	AP	b2.4	0.24	0.10	0.39	0.07	0.22	< 0.01
		a1*b1.4	0.24	0.15	0.34	0.05	0.18	< 0.01
		a2*b2.4	0.23	0.09	0.38	0.07	0.17	< 0.01
		c4 + a1*b1.4	0.58	0.39	0.78	0.10	0.43	< 0.01
		c4 + a2*b2.4	0.57	0.42	0.74	0.08	0.42	< 0.01

AS = Accepting Self-perception (decentering), DP = Distanced Perspective (decentering), Se = Self-esteem, SP = self-protection, SAi = internal self-affirmation resources, SAe = external self-affirmation resources, Est = unstandardized regression coefficient, SE = standard error, b = standard regression coefficient.

### Perceived social support

Regarding hypothesis 2.1, we found that perceived social support was significantly positively predicted by self-affirmation using external resources (*β* = 0.18, SE = 0.03, *p* < 0.001) and significantly negatively by self-affirmation using internal resources (*β* = −0.10, SE = 0.04, *p* = 0.008). Hypothesis 2.1 was thus only partially confirmed. Self-enhancement did significantly positively predict perceived social support (*β* = 0.40, SE = 0.06, *p* < 0.001). For self-protection we found a significant negative prediction (*β* = −0.32, SE = 0.06, *p* < 0.001). Hypothesis 2.2 and 2.3 could thus both be confirmed.

### *Post-hoc* network analysis

**Figure 2:**
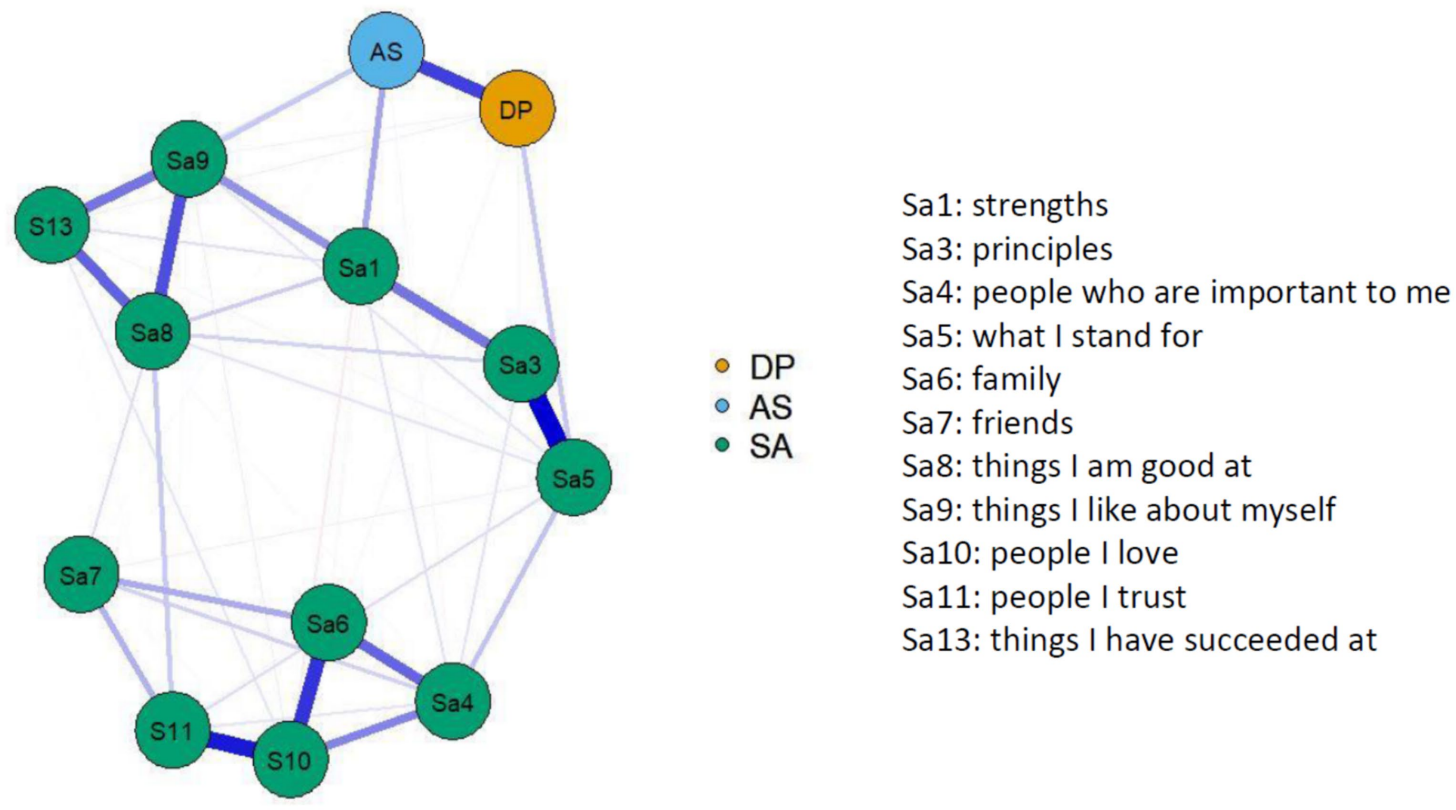
Network plot with decentering factors and self-affirmation items.

The network analyses were conducted using the mean scores of the two decentering factors, *Distanced Perspective* and *Accepting Self-Perception*, and the individual strategies. The network with decentering and self-affirmation is shown in Figure 2. Each figure also includes an abbreviated description of the respective questionnaire items which is intended to simplify the substantive interpretation of the network. The fully formulated questionnaire items can be found in Supplementary Table S2. Figure 2 shows that the two decentering factors are highly correlated with each other. Among the self-affirmation strategies, item 8, 9, and 13, all of which relate to personal achievements (see Figure 2), item 3 and 5 (personal principles), and item 4, 6, 10, and 11 (important others) are the most highly correlated. The self-affirmation items most closely associated with decentering, especially *Accepting Self-Perception*, are item 1 (strengths) and 9 (things I like about myself). Additionally, the centrality measures, strength and betweenness, were calculated for each network. The figures for these analyses can be found in the Supplementary Figures S1–S6. The highest strength (sum of the total number of connections a node has in a network) is shown by self-affirmation item 10 (people I love), followed by 8 (things I am good at) and 9 (things I like about myself) (Supplementary Figure S1). The highest betweenness (how often a node lies on the path between two other nodes, thus acting as a mediator) is shown by self-affirmation item 5 (what I stand for), followed by 4 (people who are important to me) and 9 (things I like about myself) (Supplementary Figure S2).

**Figure 3:**
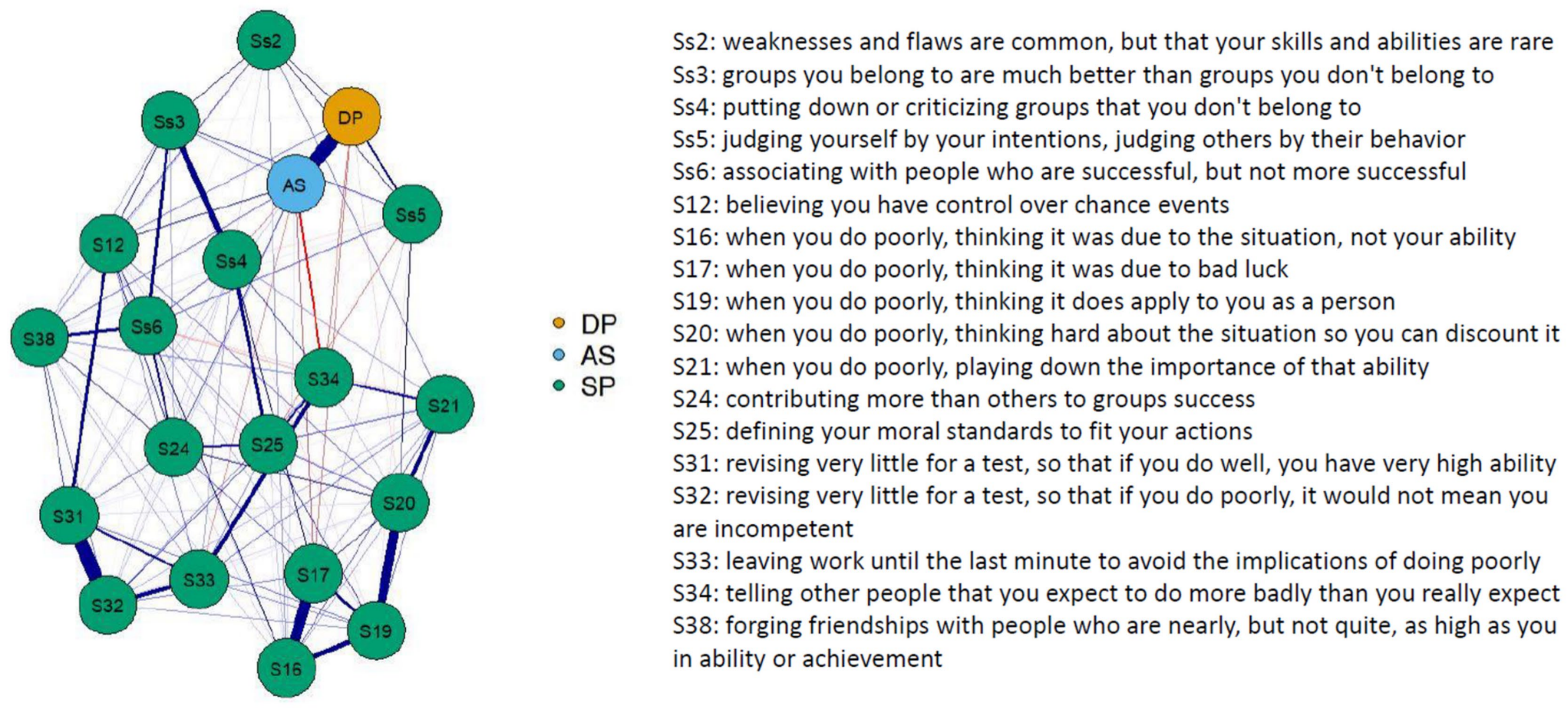
Network plot with decentering factors and self-protection items.

In the network with self-protection, items 3 and 4 (favoring one’s own group and talking down other groups) are highly correlated with each other. Item 4 shows a negative correlation with *Accepting Self-Perception*. Furthermore, item 16, 17, and 19 (evaluating one’s ability in case of failure) and item 31, 32, and 33 (preparing little for an exam to avoid attributing failures to one’s abilities) are highly correlated with each other (Figure 3). The decentering factors *Accepting Self-Perception*, as well as *Distanced Perspective*, show a strong negative correlation with item 34 (self-handicapping). Item 34 is also negatively correlated with items 17, 5, and 6 (Figure 3). Otherwise, it is noticeable that most self-protection items seem to be positively correlated with *Accepting Self-Perception*. The highest strength was shown by items 19, 20, 31, and 32 (Supplementary Figure S3), and the highest betweenness was shown by item 20, *Accepting Self-Perception*, and item 34 (Supplementary Figure S4).

**Figure 4:**
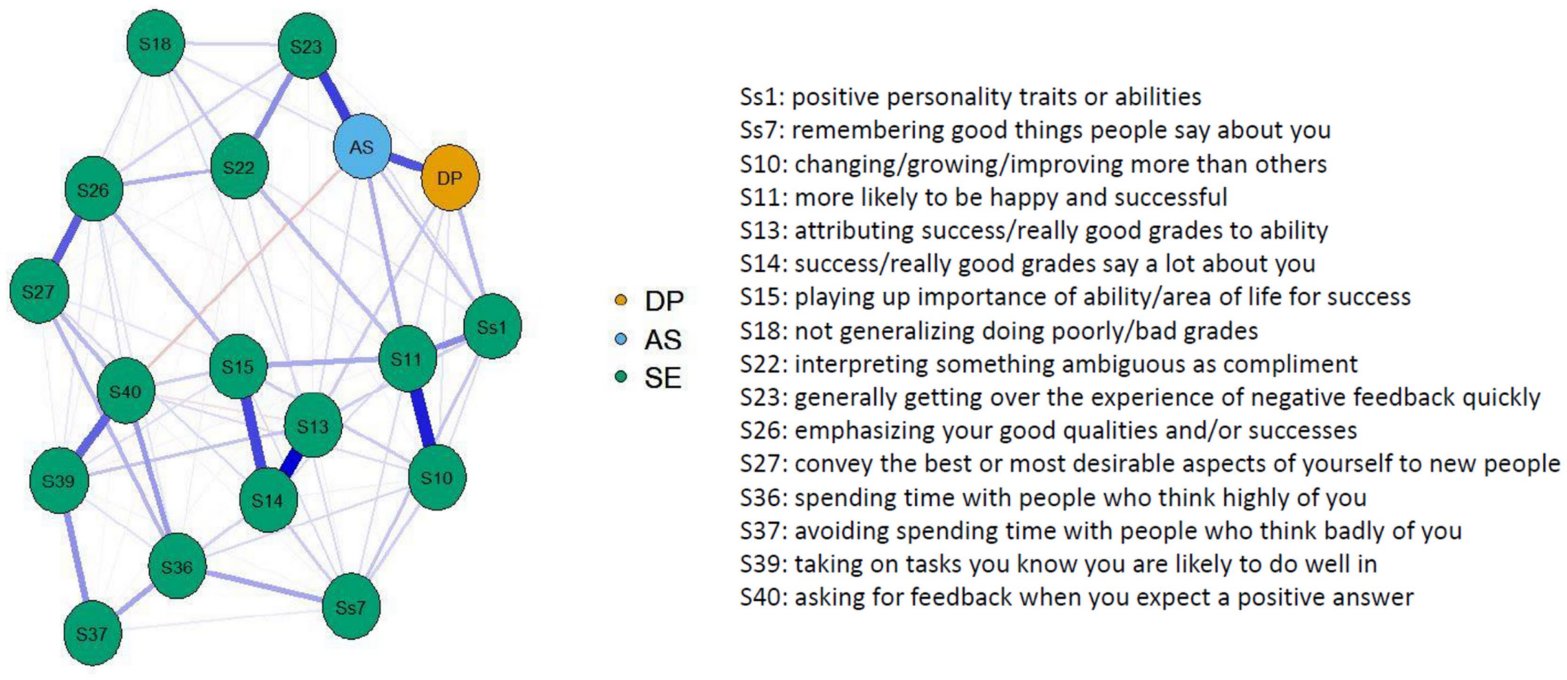
Network plot with decentering factors and self-enhancement items.

Lastly, the network with the self-enhancement strategies again shows a high correlation between the two decentering factors, as well as between *Accepting Self-Perception* and item 23 (getting over the experience of negative feedback quickly), item 23 and item 22 (interpreting something ambiguous as a compliment), item 10 (growing more than others) and item 11 (more likely to be happy and successful), items 13, 14, and 15 (internal attribution of success), items 26 and 27 (showing oneself in the best light), and items 39 and 40 (fishing for compliments). Interestingly, *Accepting Self-Perception* negatively correlates with item 40 (Figure 4). Regarding the centrality measures, items 11, 14, and *Accepting Self-Perception* show the highest strength. *Accepting Self-Perception* also has the highest betweenness.

## Discussion

The aim of the present study was to investigate factors influencing the use of self-esteem strategies, as some strategies are considered more adaptive (such as self-affirmation) in terms of open-mindedness while others are considered more maladaptive (such as self-protection) in terms of defensiveness. In addition to the well-studied influence of trait self-esteem, decentering was examined as a factor contributing to inter-individual variability in the use of regulative strategies. It was hypothesized that decentering would facilitate positive self-focused attention and better access to one’s own resources, thereby enabling the use of self-affirmation strategies. Decentering was thus explored as a mediating process between self-esteem and the strategies of self-affirmation, self-enhancement, and self-protection. In the correlation analyses, we were able to replicate the positive association between self-esteem and decentering (Mainz et al., 2016; Rader et al., 2023) as well as partially replicate the well-known associations between self-esteem and the strategies (e.g., Düring and Jessop, 2015; Harris et al., 2019; Hepper et al., 2010). Furthermore, we gained initial insights into the relationships between decentering and the strategies. Based on previous research findings, we expected decentering to correlate most strongly with self-affirmation, followed by self-enhancement, and that self-protection would negatively correlate with decentering. When looking at the correlations between the questionnaire means, this pattern largely holds true. Specifically, internal self-affirmation resources (strengths and values) correlate highly with decentering. The structural equation model showed a slightly more complex picture, as the explained variance of all variables was considered in this model. Both decentering factors (*Accepting Self-perception* and *Distanced Perspective*) were significant mediators in the relationship between self-esteem and self-affirmation through *internal resources* (e.g., personal strengths and values). For self-affirmation using *external resources* (e.g., friends, family), only the *Distanced Perspective* factor was a significant mediator. The relationship between self-esteem and self-enhancement was significantly mediated by both decentering factors. In the case of self-protection, a significant mediation through *Accepting Self-perception* was also found; however, contrary to expectations, a positive rather than a negative relationship between self-protection and *Accepting Self-perception* was observed. This result was contrary to our expectations, as self-protection is described in the literature as a rather dysfunctional strategy in the long-term that is negatively related to self-esteem (e.g., Harris et al., 2019), which in turn is positively related to decentering (Mainz et al., 2016; Rader et al., 2023). Therefore, a negative relationship between self-protection and decentering was expected. The correlation analyses showed a weak but significantly positive correlation between self-protection and both decentering factors. On the other hand, self-protection was the only strategy that correlated significantly positively with rumination, which has been contrasted to decentering by some researchers (Fresco et al., 2007a; Fresco et al., 2007b). For example, rumination is a core symptom of depression and is positively associated with it (Spasojević and Alloy, 2001), while decentering is negatively correlated with depression (Bennett et al., 2021). However, the network analysis showed that the self-protection item 34 and *Accepting Self-perception* are, for example, negatively associated. It thus appears that there are more or less dysfunctional self-protection strategies, with for example self-handicapping (telling other people that you expect to do worse than you actually do, item 34) seeming to be a rather more dysfunctional strategy.

The dysfunctionality of self-protection is moreover evident in its significant negative relationship with perceived social support. One possible explanation for this negative link could be that people with high self-protection tendency prefer to solve conflicts by themselves and reject support from their social environment which might cause the environment to refrain from offering support to the person in the long-term. An alternative explanation could be that people with a low self-esteem and a high self-protection tendency might have a distorted perception in a way that they have the feeling that they are less accepted by their environment (e.g., like a miscalibrated sociometer). This subjective perspective might, however, not be congruent with the perception from the outside. A study by Murray et al. (2008) found competing goals between self-protection and connectedness in people with low self-esteem. They assume that people with low self-esteem prefer self-protection over connectedness with their romantic partners and show low-risk behavior that directs them away from situations where they need to trust and depend on others (Murray et al., 2008). Similarly, Shaver et al. (2017) postulated a link between anxious attachment styles and self-protection strategies. They describe attachment-related avoidance as the “extent to which a person distrusts others’ good will and defensively strive to maintain behavioral and emotional self-protection and independence” (Shaver et al., 2017, p. 3). They further state that insecure or anxious attachment is associated with lower other-compassion and interferes effective care-giving (e.g., defensive reactions to others’ needs; Shaver et al., 2017). In summary, it could be assumed that a cognitive bias in the perception of social support among individuals with low self-esteem leads to an anxious attachment style and a heightened need for self-protection. Defensive behaviors and a possibly reduced other-compassion, in the long run, lead to an interaction with the social environment, potentially causing the social environment to actually turn away from the individual which in our view underlines the dysfunctionality of this strategy.

The idea, to examine self-affirmation, self-enhancement and self-protection strategies for self-esteem regulation is based on a study by Tesser et al. (2000) amongst others. They found that people seem to be self-esteem satisficers rather than maximizers. When the participants’ self-esteem was already enhanced by one strategy such as self-affirmation, they did not feel the need to use another strategy (such as self-enhancement or self-protection) to enhance their self-esteem even more (Tesser et al., 2000). This idea is in line with the negative feedback loop proposed by the sociometer theory – people just need to enhance their self-esteem until their individual sense of belonging is restored and there is no reason to further increase self-esteem (Leary, 2005). Therefore, the current study aimed to examine processes such as decentering that could be used to specifically promote the use of self-affirmation strategies which has been described as a functional self-esteem strategy so that people might not feel the need to resolve to other strategies like self-protection. However, in contrast to the results by Tesser et al. (2000) the results of the current study suggest that people with higher self-esteem use generally more strategies than people with low self-esteem (see Supplementary Table S1). This would implicate that people with high self-esteem seem to be self-esteem maximizers whereas people with low self-esteem seem to be motivated to consolidate their low self-esteem which is a finding that has also been found in previous studies (Mahadevan et al., 2016; Wright et al., 2014). Mahadevan et al. (2016) for instance found a positive association between affiliative behavior and trait self-esteem. They state that based on the sociometer theory one would expect a negative association – the lower the self-esteem the more affiliative behavior would be expected in order to restore the sense of belonging and compensate or regulate the lowered self-esteem. Instead, people with low self-esteem showed avoidant and consolidating behavior (Mahadevan et al., 2016). This finding could be explained through the concept of self-continuity. In addition to the motive of self-enhancement, the motive of continuity can be particularly relevant for identity formation as well as experience and behavior (Vignoles et al., 2006). People with low self-esteem might therefore feel less need to regulate their self-esteem after a negative event such as criticism or rejection, as they may perceive this as incongruent with their identity. Thus, people with low self-esteem are more likely to accept negative feedback compared to those with high self-esteem or may have more difficulty accepting compliments. One strategy that fits this is self-verification (Swann, 2012). For example, studies with patients with depression have already shown that people with very low self-esteem experience negative feedback as more congruent than people with high self-esteem (Giesler et al., 1996). Again, these findings could possibly be explained by a chronically miscalibrated sociometer which might lead distorted processing of information (Murray et al., 2002). Consequently, people with low self-esteem may feel threatened more easily but lack access to their resources, thus resorting to rather maladaptive self-esteem regulation strategies such as self-protection. As a result, their self-esteem cannot stabilize or increase in the long term. It is further assumed that this process may become a chronic one over the years, with cognitive distortions and defensive behavior patterns becoming entrenched due to a lack of functional alternatives. In the present study, it was shown in a healthy sample that self-esteem regulation generally decreases with lower self-esteem, though a certain degree of regulation or protection of self-esteem still seemed to be predominantly present. However, once this regulation diminishes beyond a certain point, for instance, when individuals no longer protect their self-esteem after criticism or rejection but instead perceive it as congruent with their self-image in terms of self-continuity or self-verification, this might be considered a pathological level that could be observed in patients with depression, for example (e.g., Giesler et al., 1996).

The results of the present study thus also suggest that the sociometer theory only seems to explain normal or healthy self-esteem regulation. In individuals with low self-esteem, up to a pathological level as seen in people with depression, other motives, such as self-continuity, appear to come into play. An alternative explanation is that people with low self-esteem may eventually stop regulating their self-esteem because they simply lack access to their resources. The finding that self-protection is most commonly used by people with low self-esteem even though people with low self-esteem generally seem to use fewer strategies and that self-protection, in turn, was to our surprise positively correlated with accepting self-perception, could suggest that, for people with low self-esteem, strengthening accepting self-perception should be the first step. This would enable them to utilize self-protection as a means of regulating their self-esteem. Over time, a more differentiated self-esteem regulation should be promoted through the distanced perspective, where self-affirmation strategies are used more frequently, and self-protection and self-enhancement strategies are used less. This idea can be well integrated into research from Sherman (2013) and Critcher and Dunning (2015) who postulated that self-affirmation works by boosting one’s own resources, broadening one’s perspective, and ultimately decoupling the self from the threat. This described broadening of one’s perspective corresponds to the *Distanced perspective* aspect of decentering postulated by Gecht et al. (2014) and Rader et al. (2023), which also involves perceiving one’s own thoughts and feelings without judgment. The detachment of the threat from the self – for instance, not taking criticism personally – could thus be facilitated by decentering.

As self-esteem represents a transdiagnostic factor and self-esteem regulation can be impaired in many different mental disorders such as depression, eating disorders, substance use disorders, or personality disorders (Colmsee et al., 2021; Ersöğütçü and Karakaş, 2016; Kresznerits et al., 2022; Orth and Robins, 2013; Winter et al., 2018), we would like to shortly discuss the implications of the results for individuals with mental health disorders. Lots of studies have already shown that decentering is a metacognitive process that can be learned and trained. There are many interventions focused on strengthening decentering through mindfulness training, such as Mindfulness-Based Cognitive Therapy (MBCT, Segal et al., 2018) or Mindfulness-Based Stress Reduction (MBSR, Kabat-Zinn, 2003). There are studies that have shown that decentering contributes to symptom reduction (e.g., less rumination; Bennett et al., 2021). The findings of the present study could be used to incorporate decentering more deliberately into therapy and, by strengthening decentering, enhance access to one’s own resources, thereby promoting a more functional and sustainable regulation of self-esteem. Acceptance and Commitment Therapy (ACT, Hayes et al., 2012), for instance, already implements two important components for this purpose: cognitive defusion, a concept closely related to decentering, and values, which are an important aspect of self-affirmation (Bernstein et al., 2019; Bramwell and Richardson, 2018). The results of the present study suggest a potentially suitable sequence for strengthening decentering in therapy. The structural equation model demonstrated that the accepting self-perception factor positively predicted all strategies, including self-affirmation through external resources (e.g., friends, family) and, most notably, self-protection. For individuals with low self-esteem, who generally tend to use fewer strategies, this could serve as a helpful starting point to regulate their self-esteem after a threat. Once an initial stabilization of self-esteem is achieved, a subsequent step could involve strengthening the distanced perspective to work on more differentiated self-esteem regulation. This is because distanced perspective was shown to positively predict self-affirmation through internal resources and negatively predict self-protection. The role of both decentering aspects *accepting self-perception* and *distanced perspective* in self-esteem regulation in different mental health disorders should be further examined in future studies. It is also important to note that low self-esteem can play a significant role not only in pathological aspects of self-esteem regulation, e.g., in people with mental health disorders but also in normal human regulation as in people who have experienced discrimination, for example. The negative effects of stereotype threats (the awareness that one might be negatively evaluated based on one’s social identity, Steele, 1998) on mental health and performance have mainly been studied in various ethnic groups, women, and individuals who identify as part of the LGBTQAI+ community. It has also been shown that self-affirmation can buffer the negative effects of stereotype threats (e.g., Cohen and Sherman, 2014). However, it is also important to investigate how discrimination and prejudice can be reduced from the outset. Racism, derogation, and the condemnation of other groups serve to enhance one’s own self-esteem and can be considered extreme forms of self-protection to maintain self-integrity (Badea and Sherman, 2019; Fein and Spencer, 1997). It has been shown by several researchers that when a person is given the opportunity to use self-affirmation strategies it reduces prejudice against other groups (Badea and Sherman, 2019; Fein and Spencer, 1997). Future studies should investigate whether a decentering training can be equally or even more effective in reducing stereotypes and prejudices.

## Limitations

In the present study, a cross-sectional design was used to investigate the relationships between self-esteem, decentering, and strategies for the first time. However, this design does not allow for conclusions about possible causal relationships between the constructs such as the positive upward spiral through decentering and self-affirmation postulated by Sherman (2013). The correlations found here should therefore be validated using longitudinal study designs, such as ecological momentary assessment (EMA), as well as experimental designs. We would also like to point out that the participants were recruited from two Western countries (the UK and the USA), and the results may not necessarily be generalizable to other countries and cultures. For example, there is evidence that American and Chinese samples differ in terms of self-esteem, with American participants typically showing higher self-esteem (Cai et al., 2009). Although more recent findings indicate a convergence in self-esteem levels between American and Chinese participants (Li et al., 2020), it would still be interesting to examine the results of the present study in a different cultural context. As an additional limitation, we would like to point out that self-esteem was not evenly distributed in the sample, with individuals with medium and high self-esteem being overrepresented. Therefore, interpretations regarding differences between high and low self-esteem should be made with caution. The relationships examined in this study should be investigated again in a sample with greater variance in self-esteem to validate the findings of the present study.

Furthermore, it should be noted that the inconsistent findings regarding the (expected) negative relationship between self-esteem and self-protection on the one hand, and the (unexpected) positive relationship between self-protection and accepting self-perception on the other hand, could also be attributed to methodological weaknesses of the self-protection scale. The relationships between the self-protection items shown in the network analysis (Figure 3) indicate that some items correlate more strongly with each other than others, which could be due to both content overlap and the similar wording of the items. Therefore, we recommend that the relationships between decentering and, specifically, self-protection be re-examined using alternative methods. It should also be noted that the regression weight for the relationship between accepting self-perception and self-protection is relatively small and should therefore be interpreted with caution. The same applies to the relationships between the two decentering factors and self-enhancement. Here as well, a methodological review of the questionnaire would be advisable. Another limitation could be the use of the EQ to measure decentering since the EQ has been partially criticized for capturing not only decentering, which is primarily assessed with the *Distanced perspective* factor, but also self-compassion (*Accepting self-perception*), which Hadash et al. (2017) define as a separate construct. On the other hand, the use of the EQ and the *Accepting self-perception* factor has provided evidence that self-compassion seems to play an important role in self-esteem regulation alongside decentering. Therefore, future studies should also measure self-compassion in addition to decentering. For future research, we also recommend a multimethod approach, using several questionnaires to assess decentering, as psychological constructs are often too complex to be represented in a single questionnaire (Eid and Diener, 2006). In addition to the EQ, for example, the decentering questionnaire by Hanley et al. (2020), which was developed based on Bernstein et al.'s (2019) metacognitive process model of decentering, could be used. This questionnaire was developed as a trait and state version and might be useful for longitudinal study designs (Hanley et al., 2020). The use of this questionnaire would also make it possible to examine the relationships between the three components of the meta-cognitive process model – *meta-awareness, disidentification from internal experiences,* and *reduced reactivity to thought content* – and self-esteem regulation, about which we currently know very little. It could be hypothesized that the third component, in particular, is strongly related to not taking criticism personally and not responding defensively to it. When comparing Bernstein et al.’s (2015) meta-cognitive decentering model with the data-driven two-factor decentering model, the greatest overlaps could be expected between Bernstein’s *disidentification from internal experience* and the *Distanced Perspective* factor, as well as between Bernstein’s *reduced reactivity to thought content* and the *Accepting Self-perception* factor, while *meta-awareness* is given little consideration in the two-factor model. In particular, the network analysis conducted in the present study suggests that *Accepting Self-perception* plays a crucial role in self-esteem regulation and is linked to all strategies, whereas the *Distanced Perspective* is primarily associated with functional self-affirmation. This raises the question of whether *meta-awareness* plays any role in self-esteem regulation at all.

## Conclusion

The current study examined if the inter-individual variability in the use of self-esteem strategies can be explained by decentering next to self-esteem. We conducted an online study in which 1,100 participants took part. In line with previous studies we found that self-affirmation and self-enhancement were positively associated with self-esteem. In the structural equation model, self-protection was significantly negatively predicted by self-esteem. The structural equation model further showed that both decentering factors (*Distanced Perspective* and *Accepting Self-perception*) were significantly positively predicted by self-esteem. Decentering significantly mediated the association between self-esteem and the strategies. For external self-affirmation resources we found a full mediation by the decentering factor *Distanced perspective*. The association between self-esteem and internal self-affirmation resources, self-enhancement and self-protection was partially mediated by decentering. We also found a positive association between perceived social support and self-esteem which is in line with assumptions of sociometer theory. Perceived social support was positively predicted by self-enhancement and external self-affirmation resources and negatively by self-protection. The post-hoc network analyses showed that *Accepting self-perception* played a central role in all three networks. The network with self-protection also showed a more differentiated picture regarding the dysfunctionality of the strategies and its associations with decentering. The network with self-affirmation emphasized the centrality of social relations (e.g., people I love) and the awareness of one’s personal strength and their associations with decentering. The results of the present study were discussed in the light of clinical as well as societal implications.

## Data Availability

The datasets presented in this study can be found in online repositories. The names of the repository/repositories and accession number(s) can be found below: https://osf.io/62c3r/.

## Footnotes

^1^ https://osf.io/62c3r/
