# Supplementary material for: Take a step back to see your own value: on the role of metacognition in self-esteem regulation
Source: Front Psychol. 2025 Mar 14;16:1530008. doi: 10.3389/fpsyg.2025.1530008 (PMC11949939; doi:10.3389/fpsyg.2025.1530008)
Supplement: Supplementary file 1 [file Supplementary_file_1.docx]

**Supplemental Material**

Take a Step Back to See Your Own Value: On the role of Metacognition in Self-esteem Regulation

L. Rader, S. D. Forster, S. Gauggel, B. Drueke & V. Mainz

Supplements 1

Prior to estimating the structural equation model, variance analyses (ANOVAs) were conducted to explore whether there are significant differences in the dependent variables of the structural equation model (for testing hypothesis 1) across different levels of self-esteem. For this purpose, self-esteem was divided into low, medium, and high levels. As the RSES scores can range from 10 to 40, low self-esteem was defined as RSES scores from 12 to 20, medium from 21 to 29, and high from 30 to 40. The RSES groups were defined as the independent variable in the ANOVAs. Separate analyses were conducted for each outcome variable. The strategies self-affirmation (*Internal resources*, *External resources*), self-enhancement, and self-protection, the decentering factors *Accepting Self-Perception* and *Distanced Perspective*, and social support were each defined as dependent variables. For significant results, a Tukey HSD post-hoc test was performed. For each analysis, the effect size partial eta-squared (η²) was calculated. An η² = 0.01 indicates a small effect, η² = 0.06 indicates a medium effect, and η² = 0.14 indicates a large effect (Cohen, 1973).

The ANOVAs showed significant differences between different self-esteem levels in self-affirmation (*Internal resources*: *F*(2, 1097) = 190.02, *p* < .01; *External resources*: *F*(2, 1097) = 40.59, *p* < .01), self-enhancement (*F*(2, 1097) = 132.48, *p* < .01), self-protection (*F*(2, 1097) = 10.79, *p* < .01), decentering (AS: *F*(2, 1097) = 337.94, *p* < .01; DP: *F*(2, 1097) = 125.74, *p* < .01) and social support (*F*(2, 1097) = 84.95, *p* < .01). The post-hoc tests showed that the high and low self-esteem and the high and medium self-esteem groups differed significantly for all variables (except for self-protection for which all three groups differed significantly). The effect sizes were small to moderate (see Table S1).

Table S1

*Means and standard deviations of study variables for levels of low, medium and high self-esteem and results of ANOVA comparing all three groups*

|  | Low | | Medium | | High | |  |  |  |
| --- | --- | --- | --- | --- | --- | --- | --- | --- | --- |
|  | Mean | SD | Mean | SD | Mean | SD | F | p | $\eta^{2}$ |
| Self-affirmation (internal) | 21.2 | 8.73 | 30.2 | 7.48 | 35.2 | 7.66 | 190.02 | <.01 | .26 |
| Self-affirmation (external) | 21.9 | 7.67 | 24.8 | 6.29 | 27.0 | 6.15 | 40.59 | <.01 | .07 |
| Self-enhancement | 45.2 | 12.8 | 56.0 | 10.7 | 61.1 | 10.5 | 132.48 | <.01 | .19 |
| Self-protection | 44.2 | 13.4 | 49.8 | 13.7 | 47.6 | 13.9 | 10.79 | <.01 | .02 |
| Distanced Perspective | 11.4 | 2.80 | 13.6 | 2.40 | 14.9 | 2.46 | 125.74 | <.01 | .19 |
| Accepting Self-perception | 12.0 | 3.43 | 17.0 | 3.51 | 19.8 | 3.23 | 337.94 | <.01 | .38 |
| Social support | 17.6 | 6.13 | 20.5 | 5.15 | 23.2 | 4.42 | 84.95 | <.01 | .13 |

*Note*. Low = low self-esteem (RSES 12-20; n = 171); Medium = medium self-esteem (RSES 21-29; n = 468); High = high self-esteem (RSES 30-40; n = 461); SD = Standard deviation; F = F-statistic of ANOVA; p = p-value of ANOVA F-test; η^2^ = effect size partial eta-squared of ANOVA F-test

Table S2

*Questionnaire items used in the current study*

| Questio-nnaire | Factor | Items | |
| --- | --- | --- | --- |
| Rosenberg Self-esteem Scale | | 1 | On the whole, I am satisfied with myself. |
|  |  | 2 | At times I think I am no good at all. |
|  |  | 3 | I feel that I have a number of good qualities. |
|  |  | 4 | I am able to do things as well as most other people. |
|  |  | 5 | I feel I do not have much to be proud of. |
|  |  | 6 | I certainly feel useless at times. |
|  |  | 7 | I feel that I'm a person of worth, at least on an equal plane with others. |
|  |  | 8 | I wish I could have more respect for myself. |
|  |  | 9 | All in all, I am inclined to feel that I am a failure. |
|  |  | 10 | I take a positive attitude toward myself. |
| Experiences Questionnaire | Accepting Self-perception | 3 | I am better able to accept myself as **I** am. |
|  |  | 5 | **I** am kinder to myself when things go wrong. |
|  |  | 6 | I can slow my thinking at times of stress. |
|  |  | 8 | **I** am not so easily carried away by my  thoughts and feelings. |
|  |  | 9 | **I** notice that **I** don't take difficulties so  personally. |
|  |  | 10 | I can separate myself from my thoughts and feelings. |
|  |  | 15 | I can observe unpleasant feelings without being drawn into them. |
|  | Distanced Perspective | 16 | I have the sense that I am fully aware of what is going on around me and inside me. |
|  |  | 17 | I can actually see that I am not my thoughts. |
|  |  | 18 | I am consciously aware of a sense of my body as a whole. |
|  |  | 20 | I view things from a wider perspective. |
| Spontaneous Self-Affirmation Measure | Internal resources | 2 | ***…***thinking about my values. |
|  |  | 3 | ***…***thinking about my principles. |
|  |  | 5 | ***…***thinking about what I stand for. |
|  |  | 12 | ***…***thinking about the things I believe in. |
|  |  | 1 | ***…***thinking about my strengths. |
|  |  | 8 | ***…***thinking about the things I am good at. |
|  |  | 9 | ***…***thinking about the things I like about myself |
|  |  | 13 | ***…***remembering things I have succeeded at. |
|  | External resources | 4 | ***…***thinking about the people who are important to me. |
|  |  | 6 | ***…***thinking about my family. |
|  |  | 7 | ***…***thinking about my friends. |
|  |  | 10 | ***…***thinking about the people I love |
|  |  | 11 | ***…***thinking about the people I trust |
| Self-enhancement Self-protection Scale | Self-enhancement | 1 | Thinking of yourself as generally possessing positive personality traits or abilities to a greater extent than most people |
|  |  | 7 | Remembering for a long time the good things that people say about you |
|  |  | 8 | Remembering hardships that you had to overcome in order to be really successful |
|  |  | 9 | Thinking about how you have grown and improved as a person over time; how much more good/honest/skilled you are now than you used to be |
|  |  | 10 | Believing that you are changing, growing, and improving as a person more than other people are |
|  |  | 11 | Believing you are more likely than most people to be happy and successful in the future |
|  |  | 13 | When you achieve success or really good grades, thinking it was due to your ability |
|  |  | 14 | When you achieve success or really good grades, thinking it says a lot about you as a person |
|  |  | 15 | When you achieve success or really good grades, playing up the importance of that ability or area of life |
|  |  | 18 | When you do poorly at something or get bad grades, thinking it only applies to specific aspects of your ability, not you as a person |
|  |  | 23 | When someone says something ambiguous about you, interpreting it as a positive comment or compliment (e.g., if someone says "you certainly speak your mind, don't you?", you might think they were praising your honesty, not insulting your lack of tact) |
|  |  | 24 | Generally getting over the experience of negative feedback quickly, so a few hours/days/weeks after a negative event (e.g., doing poorly in an exam, being criticized by a friend) you no longer feel bad |
|  |  | 27 | Emphasizing your good qualities and/or successes, but not your weaknesses and/or failures, when talking to new people |
|  |  | 28 | Ensuring that you convey the best or most desirable aspects of yourself to new people through your behavior (e.g., wearing clothes that give the 'right' message, expressing opinions they will approve of) |
|  |  | 29 | When you do poorly at something, reminding yourself of your other strengths and abilities |
|  |  | 30 | In times of stress, reminding yourself of your values and what matters to you |
|  |  | 31 | In times of stress, thinking about your positive close relationships and loved ones |
|  |  | 36 | Thinking about how things could have been much worse than they are (e.g., “well, at least…”; “it could be worse”) |
|  |  | 37 | Spending time with people who think highly of you, say good things about you, and make you feel good about yourself |
|  |  | 38 | Avoiding spending time with people who think badly of you, criticize you, or make you feel bad about yourself |
|  |  | 40 | Choosing to take on particular tasks because you know you are likely to do well in them or succeed easily |
|  |  | 41 | Asking for feedback when you expect a positive answer (e.g., asking a friend “Do I look ok?” when you have made a lot of effort with your appearance; approaching a senior colleague or tutor for feedback on a piece of work if you think you did well) |
|  | Self-protection | 2 | Thinking that your weaknesses and flaws are common, but that your skills and abilities are rare |
|  |  | 3 | Thinking that groups you belong to are generally much better than groups you don't belong to (e.g., sports teams or supporters, universities) |
|  |  | 4 | Putting down or criticizing groups that you don't belong to (e.g., a rival sports team or university) |
|  |  | 5 | Working out the kind of person you are by examining your intentions (e.g., "I am considerate because I think about how I can help others"), but working out other people only by examining their behavior (e.g., "She must be considerate because she helped a friend with his work") |
|  |  | 6 | Associating yourself with people who are successful – but not more successful than you |
|  |  | 12 | Believing you have control over chance events (e.g., thinking you are more likely than others to throw a 6 on a dice, thinking your personally chosen lottery numbers are more likely to win than “lucky dip” numbers) |
|  |  | 16 | When you do poorly at something or get bad grades, thinking it was due to the situation, not your ability (e.g., the exam questions were unfair or too difficult) |
|  |  | 17 | When you do poorly at something or get bad grades, thinking it was due to bad luck |
|  |  | 19 | When you do poorly at something or get bad grades, thinking that the situation or test was uninformative or inaccurate (e.g., thinking the exam was badly designed, or thinking "that can't be right") |
|  |  | 20 | When you do poorly at something or get bad grades, thinking hard about the situation and feedback until you find something wrong with it and can discount it |
|  |  | 21 | When you do poorly at something or get bad grades, playing down the importance of that ability or area of life |
|  |  | 25 | When a group you are part of does well, thinking that you contributed to the success more than other members |
|  |  | 26 | Defining your moral standards to fit your actions (e.g., believing that it’s ok to cheat in a game of cards, keep the extra change the cashier mistakenly gave you, or gossip about an acquaintance, because…) |
|  |  | 32 | Revising very little for a test, or going out the night before an exam or appraisal at work, so that if you do well, it would mean you must have very high ability |
|  |  | 33 | Revising very little for a test, or going out the night before an exam or appraisal at work, so that if you do poorly, it would not mean you are incompetent |
|  |  | 34 | Leaving work until the last minute (and often not getting it done) to avoid the implications of doing poorly |
|  |  | 35 | Telling other people that you expect to do even more badly than you really expect to do (e.g., in work or a sporting event) |
|  |  | 39 | Forging friendships with people who are nearly, but not quite, as high as you in ability or achievement |
| F-Sozu K6 | | 1 | I experience a lot of understanding and security from others. |
|  |  | 2 | I know a very close person whose help I can always count on. |
|  |  | 3 | If necessary, I can easily borrow something I might need from neighbours or friends. |
|  |  | 4 | I know several people with whom I like to do things. |
|  |  | 5 | When I am sick, I can without hesitation ask friends and family to take care of important matters for me. |
|  |  | 6 | If I am down, I know to whom I can go without hesitation. |

Table S3

*Allocation of items to latent factors in the structural equation model (hypothesis 1)*

| Variable name | Allocated items | Explanation |
| --- | --- | --- |
| dec1 | 16, 17, 18, 20 | Distanced Perspective factor of the EQ |
| dec2 | 3, 5, 6, 8, 9, 10, 15 | Accepting Self-perception factor of the EQ |
| values | 2, 3, 5 | Values factor of the SSAM by Harris et al. (2019) |
| strengths | 1, 8, 9, 13 | Strengths factor of the SSAM by Harris et al. (2019) |
| internal | values + strengths | Internal resources factor of the SSAM by Rader et al. (2024) |
| external | 4, 6, 7, 10, 11 | External resources factor of the SSAM by Rader et al. (2024) |
| sp | 3, 4, 16, 17, 19, 20, 21, 25, 26, 31, 32, 33, 34, 38 | Self-protection subscale of the SESP |
| se | 1, 10, 11, 18, 22, 23, 7, 13, 14, 15, 26, 27, 36, 37, 39, 40 | Self-enhancement subscale of the SESP |
| rses | 1, 2, 3, 4, 5, 6, 7, 8, 9, 10 | Rosenberg Self-esteem Scale |

*Note.* EQ = Experiences Questionnaire, SSAM = Spontaneous Self-affirmation Measure, SESP = Self-enhancement Self-protection Scale


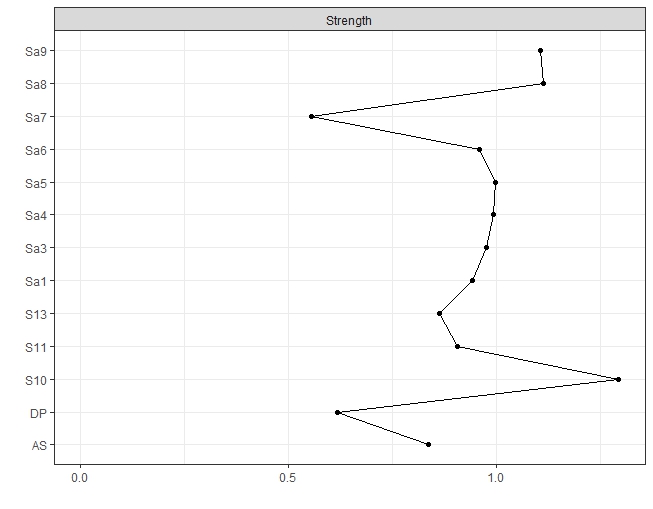


*Figure S1*. Plot of centrality measure strength of network analysis with decentering factors and self-affirmation


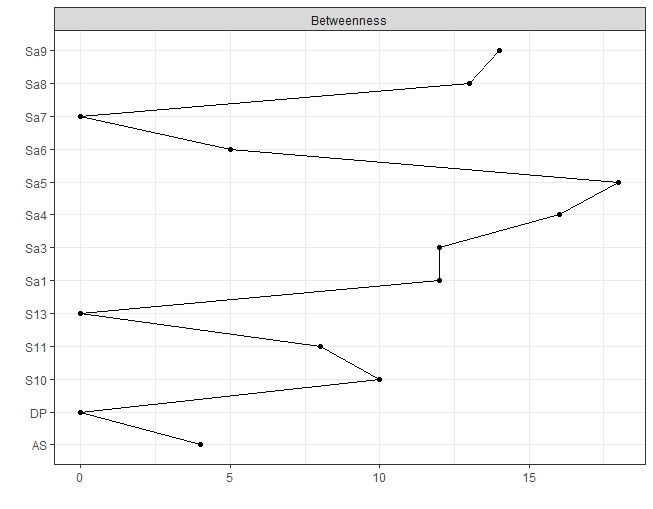


Figure S2. Plot of centrality measure betweenness of network analysis with decentering factors and self-affirmation


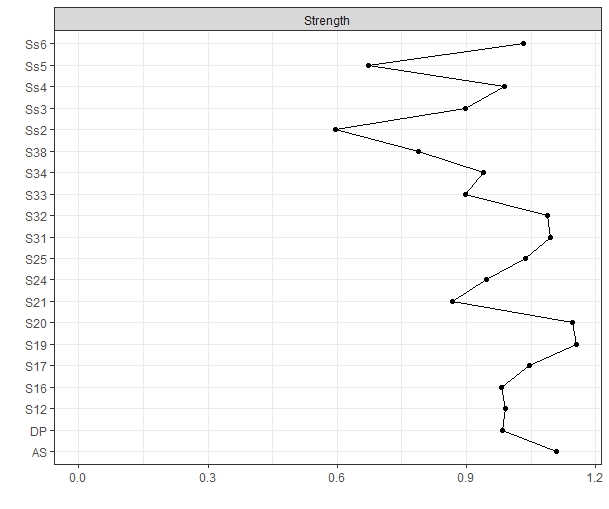


*Figure S3*. Plot of centrality measure strength of network analysis with decentering factors and self-protection


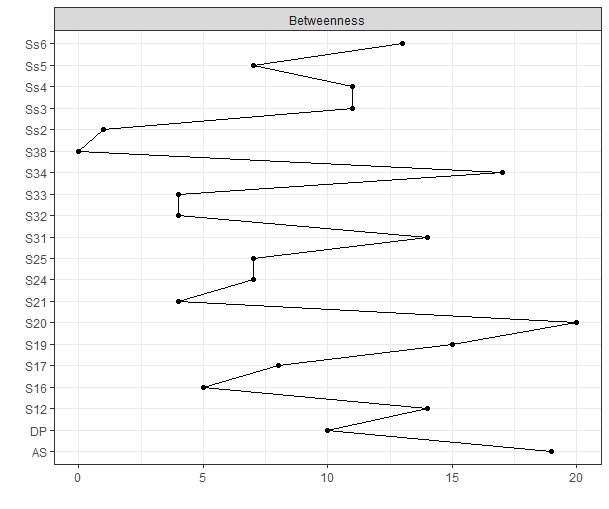


*Figure S4.* Plot of centrality measure betweenness of network analysis with decentering factors and self-protection


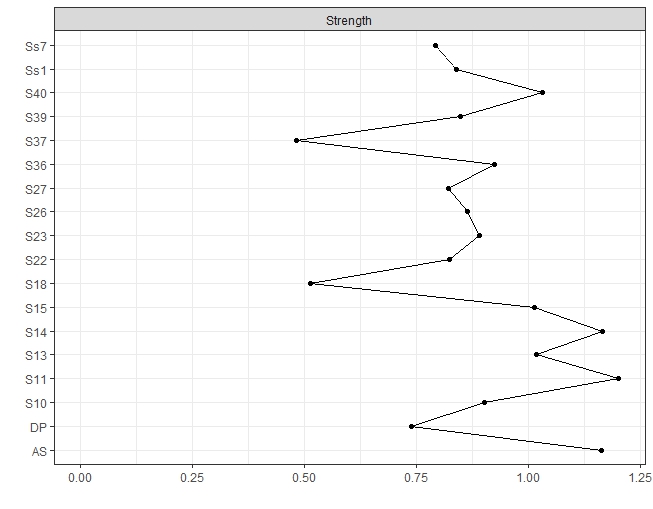


*Figure S5*. Plot of centrality measure strength of network analysis with decentering factors and self-enhancement


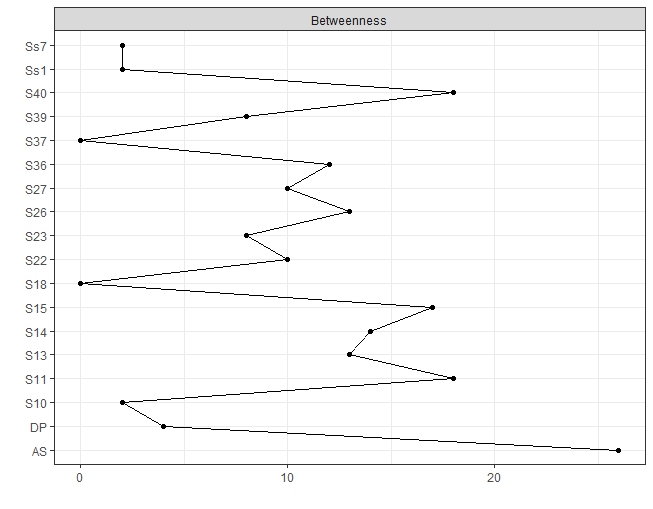


*Figure S6*. Plot of centrality measure betweenness of network analysis with decentering factors and self-enhancement
